# Artificial intelligence and social intelligence: preliminary comparison study between AI models and psychologists

**DOI:** 10.3389/fpsyg.2024.1353022

**Published:** 2024-02-02

**Authors:** Nabil Saleh Sufyan, Fahmi H. Fadhel, Saleh Safeer Alkhathami, Jubran Y. A. Mukhadi

**Affiliations:** ^1^Psychology Department, College of Education, King Khalid University, Abha, Saudi Arabia; ^2^Psychology Program, Social Science Department, College of Arts and Sciences, Qatar University, Doha, Qatar

**Keywords:** artificial intelligence, social intelligence, psychologists, ChatGPT, Google Bard, Bing

## Abstract

**Background:**

Social intelligence (SI) is of great importance in the success of the counseling and psychotherapy, whether for the psychologist or for the artificial intelligence systems that help the psychologist, as it is the ability to understand the feelings, emotions, and needs of people during the counseling process. Therefore, this study aims to identify the Social Intelligence (SI) of artificial intelligence represented by its large linguistic models, “ChatGPT; Google Bard; and Bing” compared to psychologists.

**Methods:**

A stratified random manner sample of 180 students of counseling psychology from the bachelor’s and doctoral stages at King Khalid University was selected, while the large linguistic models included ChatGPT-4, Google Bard, and Bing. They (the psychologists and the AI models) responded to the social intelligence scale.

**Results:**

There were significant differences in SI between psychologists and AI’s ChatGPT-4 and Bing. ChatGPT-4 exceeded 100% of all the psychologists, and Bing outperformed 50% of PhD holders and 90% of bachelor’s holders. The differences in SI between Google Bard and bachelor students were not significant, whereas the differences with PhDs were significant; Where 90% of PhD holders excel on Google Bird.

**Conclusion:**

We explored the possibility of using human measures on AI entities, especially language models, and the results indicate that the development of AI in understanding emotions and social behavior related to social intelligence is very rapid. AI will help the psychotherapist a great deal in new ways. The psychotherapist needs to be aware of possible areas of further development of AI given their benefits in counseling and psychotherapy. Studies using humanistic and non-humanistic criteria with large linguistic models are needed.

## 1 Introduction

Machines have influenced human evolution. The characteristics of each era have been shaped by the tools developed since the First Industrial Revolution (1760–1840), for example, the use of steam machines instead of manual labor, and the Second Industrial Revolution (1870–1914), represented by the use of energy. The use of electricity instead of steam power led to the Third Industrial Revolution (1950–1970), where electronic and communication devices such as computers and portable devices appeared. Today we are in the Fourth Industrial Revolution, which has witnessed the introduction of artificial intelligence in many fields, including health care, psychotherapy, and more (Hounshell, 1984; Mokyr and Strotz, 1998; Brants et al., 2007; Bell, 2019; Thirunavukarasu et al., 2023).

In psychotherapy, the early Eliza program, designed in the 1970s by Weitz Naum, a professor at the Massachusetts Institute of Technology, was a very primitive program, compared to the programs we see today. The program was distinguished by providing some comfort for postgraduate students. Some of them even liked to sit alone next to the computer, and found that the Eliza program helped them a lot, even though they knew it had no emotions, care, or empathy (O'Dell and Dickson, 1984).

On November 22, 2022, ChatGPT-3 became available to the general public. It was a surprise to the technological community and the world, and it was a powerful leap in the field of AI. AI is one of the most advanced areas of modern technology. It was followed by the most famous ChatGPT-4, which is nearly 500 times larger in terms of capacity and also processing capacity. It is the latest version of ChatGPT, launched in March 2023. This is a chatbot that belongs to linguistic artificial intelligence and uses artificial intelligence technology to interact with users in different languages. It has the ability to understand, create, analyze and edit texts, and uses more than 500 billion words from various sources to understand and create texts in smart and creative ways.

Companies then competed to produce large language models in AI: “LLMs.” It is an abbreviation of the term “Large Language Models,” which refers to AI models that are trained on large amounts of text for the purpose of understanding and generating natural language in an advanced way. Examples include the ChatGPT-3 and 4 from OpenAI, the LaMDA and PaLM models from Google (the basis for Bard), the BLOOM model and XLM-RoBERTa from Hugging Face, and the NeMO model From Nvidia, XLNet, Co:here, and GLM-130B.

Google Bard is a Large Language Model (LLM) created by Google AI. This is a machine-learning model trained on a huge dataset of text and code amounting to 1.56 trillion words. It can generate human-quality text, translate languages, write different types of creative content, and answer questions in a human-like manner. It first appeared on January 18, 2023, when it was announced at the Google AI Conference, and was released to the public on October 16, 2023. Bing AI Chat is a service provided by Microsoft that uses artificial intelligence to improve the search experience of users. Users can interact with Bing as if they were talking to another person, with Bing answering questions and providing information in a natural and friendly way. In addition, Bing can generate images directly from the user’s words.

This field has witnessed many important developments in recent years, and it is expected that it will continue to develop in the future at a faster rate and with greater leaps. The AI models allow machines to perform advanced human-like functions. This development began in the 1950s, and continued at varying rates until 2022, when deep learning, a branch of AI, became important in many practical applications such as image recognition and translation (Brants et al., 2007; Bell, 2019; Thirunavukarasu et al., 2023).

The mechanism used in ChatGPT-3 announced by Open AI was a breakthrough that resulted in an artificial intelligence program that can simulate human conversation. Since then, competition has flared among the major companies that had been preparing for such a day for years but were unable to launch a similar produce, namely, Microsoft and Google. Google Barge, Bing, and others introduced large linguistic conversation models that used natural human language relying on a large database; these were trained by interacting with people in specialties and in many fields, including the therapeutic psychological field (Hagendorff and Fabi, 2023; Han et al., 2023).

AI is classified into several categories according to the application, field, and techniques used. In general, it is divided into two types: weak, which is designed to perform a specific task such as voice recognition, and strong, which aims to imitate human intelligence in general (Russell and Norvig, 2010).

This year, large language models have evolved a lot and have reached a stage where they demonstrate human-like language understanding and generation capabilities, which in turn opens new opportunities for using measurement tools to identify the hidden values, attitudes, and beliefs that are encoded in these models. The capabilities of AI to diagnose personality traits and understand feelings and thoughts have been measured and their credibility has been verified by a number of studies (Maksimenko et al., 2018; Kachur et al., 2020; Flint et al., 2022; Han et al., 2023; Landers and Behrend, 2023; Lei et al., 2023; Zhi et al., 2023).

One of the contemporary studies that was concerned with measuring the capabilities of ChatGBT is the study that was presented in the technical report issued by OpenAI on March 27, 2023, in which it conducted tests similar to admission tests in various professional and academic American universities. It included the SATs, the Bar Exam, and the AP final exams. The results showed that the ChatGPT 3.5 and ChatGPT 4.0 are capable of performing human-like on many professional and academic tests.

### 1.1 Artificial intelligence in psychotherapy field

When a psychologist or counselor carries out the counseling and psychotherapy process, they go through several stages that starting with the preparation phase, which requires several skills, including social intelligence skills. The psychologist employs these skills effectively from the first session and continues until the closing of the sessions. For this reason, previous psychological studies have examined the capabilities of artificial intelligence systems, especially linguistic models, in the therapeutic process. The research is summarized follows:

In the field of diagnosis, artificial intelligence can help improve psychological treatment by providing tools and techniques that help stimulate the process of change and focus on cognitive and emotional understanding (de Mello and de Souza, 2019). It can also contribute to measuring mental (Lei et al., 2023) and emotional disorders and thus reduce the potential risk of suicide (Morales et al., 2017; Landers and Behrend, 2023).

AI can also help improve empirical analysis by developing data-driven models and tools to address new means of selecting therapeutic models (Horn and Weisz, 2020). It can also use speech content analysis and measure mental and emotional disorders as well as the effect of psychiatric medications (Gottschalk, 1999). In addition, AI can use the analysis of physiological signals such as pulse rate, galvanic skin response, and pupil diameter to monitor stress level in users (Zhai et al., 2005).

According to Kachur et al. (2020), AI has ability in the diagnostic process to accurately determine personality traits and has made multidimensional personality profiles more predictable. In another study, Maksimenko et al. (2018) found a relationship between EEG recordings and mental abilities and personality traits. They concluded the importance of designing artificial intelligence programs for personality testing that combine simple tests and EEG measurements to create accurate measurements. Kopp and Krämer (2021) evaluate the ability of intelligent models to visualize and understand mental states speaker and generate behaviors based on them. They concluded that it is necessary to use empathy and positive interactions to support understanding of silent clients.

Regarding the use of smart systems in counseling and psychotherapy, Das et al. (2022) found the effectiveness of GPT2 and DialoGPT in psychotherapy and how the linguistic quality of general conversational models improved through the use of training data related to psychotherapy. Eshghie and Eshghie (2023) showed the ability of ChatGPT to engage in positive conversations, listen, provide affirmations, and introduce coping strategies. Without providing explicit medical advice, the tool was helped therapists make new discoveries.

Likewise, a study of Ayers et al. (2023) evaluated ChatGPT’s ability to provide high-quality empathetic responses to patients’ questions and found that residents preferred chatbot answers to physician answers. Chatbot responses were rated as more empathetic than doctors’ responses. A recent study (Sharan and Romano, 2020) indicated that AI-based methods apply techniques with great efficiency in solving mental health difficulties and alleviating anxiety and depression.

Although previous studies were enthusiastic and tended to support the capabilities of artificial intelligence, there is, in contrast, an opposing view citing errors resulting from AI models in the field of mental health practices. Elyoseph and Levkovich (2023) to compare mental health indicators as estimated by the ChatGPT and mental health professionals in a hypothetical case study focusing on suicide risk assessment. The results indicated that ChatGPT rated the risk of suicide attempts lower than psychologists. Furthermore, ChatGPT rated mental flexibility below scientifically defined standards. These findings have suggested that psychologists who rely on ChatGPT to assess suicide risk may receive an inaccurate assessment that underestimates actual suicide risk.

In addition, research tended to warn against excessive confidence in these systems. Grodniewicz and Hohol (2023) investigate three challenges facing the development of AI systems used in providing psychotherapy services, and explore the possibility of overcoming them: the challenges of deep understanding of psychotherapy strategies, establishing a therapeutic relationship, and the complex voice conversation techniques compatible with humans who convey emotions in their precise structures. The benefits and side effects of using AI in the psychological field should be clarified. Chang et al. (2023) concluded that it is necessary to focus on evaluating the performance of these models, including general performance, response to a task, output, and presentation; their results were heterogeneous in output. Likewise, Woodnutt et al. (2023) found that ChatGPT was able to provide a plan of care that incorporated some principles of dialectical behavioral therapy, but the output had significant errors and limitations, and therefore the potential for harm was possible. Others have pointed out the need to treat AI as a tool but not as a therapist, and limit its role in the conversation to specific functions (Sedlakova and Trachsel, 2023). In addition, there are many challenges that must be overcome before AI becomes able to provide mental health treatment. It is clear that more research is needed to evaluate artificial intelligence to consider how it can be used safely in health care delivery (Grodniewicz and Hohol, 2023). This is why there was an urgent need to conduct this study, which aimed to identify the level of social intelligence of linguistic artificial intelligence models “ChatGPT-4; Bard; Bing” and compare it with psychologists (Bachelor’s and Doctorate holders) to reveal the extent to which artificial intelligence contributes to psychotherapy and counseling and to provide comparisons with psychologists.

Consequently, the current study examined the level of social intelligence of artificial intelligence models compared to the performance of psychologists, by using a scale designed to evaluate human social intelligence.

## 2 Methods

### 2.1 Participants and procedure

The Human participants were a sample of male psychologists in the Kingdom of Saudi Arabia with one of two levels of education (Bachelor’s and doctoral students) at King Khalid University during 2023–2024. The study sample consisted of 180 participants, including 72 bachelor’s students and 108 doctoral students in counseling psychological program. They were random selected using stratified method to fit the distribution of participants into two different educational stages. The age of the doctoral students ranged between 33 and 46 years (40.55 ± 6.288), while it was ranged between 20 and 28 years (22.68 ± 7.895) among the bachelor’s students.

In this study, a registered version of ChatGPT-4 (OpenAI, 2023) and the free version of Google Bard, and Bing were used. We conducted a single evaluation for each AI model on August 1, 2023 of its SI performance using the Social Intelligence Scale (Sufyan, 1998). In each evaluation, we provided AI the same 64 standard SI scenarios. A link to the questionnaire was sent to human participants via e-mail. While the large linguistic models of AI were asked to answer the scale items individually and their answers were collected in a separate external file by directing a question to the AI models to choose the appropriate answer from the alternative points for each item in the scale.

### 2.2 Study tools

The performance of the AI models and psychologists was scored using the standard manual (Sufyan, 1998) The SI Scale was prepared by Sufyan (1998) in Arabic to assess SI among adults in similar to the George Washington University Brief Scale of SI. It consists of 64 items and contained two dimensions: Soundness of judgment of human behavior, which represents the ability to understanding social experiences by observing human behavior. The second dimension assess the ability to act in social situations by analyzing social problems and choosing the best appropriate solutions to them. Sufyan (1998) verified the validity and reliability of this scale. However, the authors of the current study verified the psychometric properties of the scale and its suitability for the objectives of the present study, especially since it will be used to evaluate the performance of large linguistic models on social intelligence skills. Therefore, the scale was presented here to 10 psychology professors at Taiz and King Khalid Universities, and all items were approved, with some items being modified. The modifications of the scale by experts were minor and did not affect the content of the items. Items (1, 7, 12, and 23) were modified grammatically in accordance with the rules of the Arabic language without causing any change in the content of the item.

The validity and reliability sample consisted of 90 individuals from the same research community. Construct validity was verified by examining the correlations between item scores and the total score on the scale using (point, biserial) coefficient. The correlation coefficients ranged between (0.39–0.48) and were significant at the 0.05 level. Construct validity was verified by identifying the significant correlation between the dimensions scores and the total score on the scale using the Pearson correlation coefficient.

The correlation coefficient of the first dimension was 0.82 and in the second dimension, it was 0.73. The reliability of the scale was verified using the re-test method by selecting a sample of 20 undergraduate students from the same research community, and the test was re-tested after 1 month. The reliability coefficient after correction with Spearman’s equation was 0.67 for the first dimension and 0.69 for the second dimension, while the overall reliability coefficient was 0.77.

### 2.3 Scoring

The first dimension’s items (41 items) of SI scale were formulated to be answered with true or false (0–1 scores per item; range 0–41), while the answer options of the second dimension (23 items) include 4 points, three of which are false and one is correct (0–1 scores per item; range 0–23).

The total score of SI scale ranged between (0–64), with a higher score indicating higher SI. In all assessments, participants respondents from both human and nonhuman samples were asked to choose the correct answer and the higher the total score, the higher the SI. The SI results of AI models were compared with those of psychologists at both bachelors and doctoral levels.

### 2.4 Statistical analysis plan

IBM SPSS software (version 28) was used for data analysis. Independent Samples Test was used to examine test–retest reliability of the scale. The relationship between item scores and the total score on the scale was calculated using the point biserial coefficient, while the Pearson correlation coefficient was used to assess the correlation between the dimensions scores and the total score of the scale.

A one-sample *t*-test was used to compare the performance of AI models to the population represented by the psychologists; Means, standard deviations, and percentages were used to determine the ranking of AI models and psychologists.

## 3 Results

To achieve the research objectives of identifying the level of social intelligence among AI models comparing with psychologists, verification was carried out as follows:

To verify the differences between AI models and psychologists in SI, the average of SI scores for psychologists were extracted; the average scores were 39.19 of bachelor’s students and 46.73 of PhD holders. While the raw scores of the AI models were treated as representing independent individual samples (one total score for each model); the scores of SI were 59 of GPT4, 48 of Bing, and 40 of Google Bard.

Therefore, we used a one-sample *t*-test to find out whether these differences were statistically significant, as shown in Table 1.

**Table 1:** The differences between AI and psychologists in the social intelligence.

	Qualification	Mean	Standard deviation	Df	*T*	*p*-value
ChatGPT 59	Bachelor	39.19	7.927	71	21.201	0.00
	Doctoral	46.73	5.974	107	21.341	0.00
Bing 48	Bachelor	39.19	7.927	71	9.426	0.00
	Doctoral	46.73	5.974	107	2.207	0.00
Google Brand 40	Bachelor	39.19	7.927	71	0.862	0.00
	Doctoral	46.73	5.974	107	11.709	0.00

As per Table 1, the scores of the AI linguistic models are as follows: GPT 4 was 59, Bing was 48, and Google Bard was 40. There are statistically significant differences between ChatGPT-4 and Bing and the psychologists in both academic stages. The AI models have higher SI scores than the psychologists.

As for Google Bard, the result differed; its score was almost equal to that of psychologists with a bachelor’s degree, and the differences were not statistically significant. While, its differs compared to doctoral-level, whose average was higher than that of Google Bird in SI. Table 2 shows the level of social intelligence according to the percentile and the raw score for psychologists according to qualification.

**Table 2:** The level of SI among psychologists according to academic stage.

			Percentages					
		Level	5	10	25	50	75	90
Weighted average (definition 1)	SI	Doctoral	35.90	39.80	44.00	48.00	51.00	54.00
		Bachelor	24.00	25.30	34.25	40.00	46.00	48.70
Tukey’s Hinges	SI	Doctoral			44.00	48.00	51.00	
		Bachelor			34.50	40.00	46.00	

The results of this study are summarized as follows:

1. In ChatGPT-4, the score on the SI scale was 59, exceeding 100% of specialists, whether at the doctoral or the bachelor’s levels.
2. Bing, whose score on the SI scale was 48, outperformed 50% of doctoral specialists, while 50% of them outperformed him. However, Bing’s performance on the SI scale was higher than 90% of bachelor’s students.
3. Google Bard, whose score on the SI scale was (40) is superior to only 10% of doctoral holders. Interestingly, 90% of doctoral holders excelled at it. In contrast, Google Bird’s performance was higher than 50% of the specialists at the bachelor’s level, while 50% of them surpassed it, meaning that Google Bird’s performance was equal to the performance of bachelor’s students on the SI scale and the differences were not significant.

**Figure 1:**
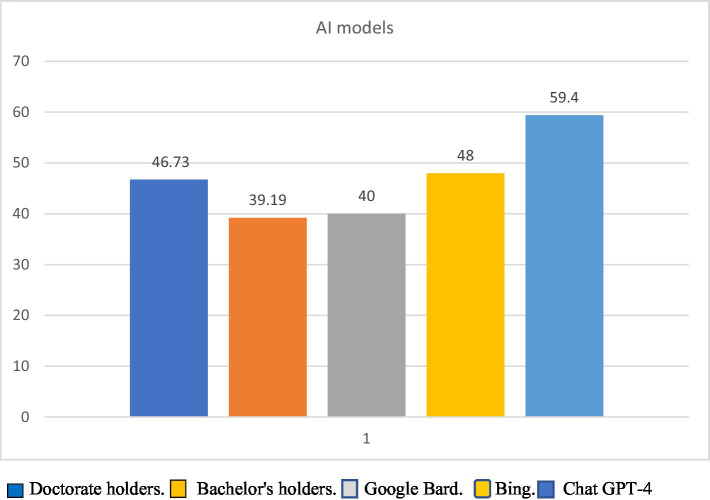
Social intelligence levels of AI models and psychologists.

Figure 1 shows SI levels of AI models and psychologists.

## 4 Discussion

The main question of this study was “Does artificial intelligence reach the level of human social intelligence?.” When we assess humans, we use psychological standards to estimate their level of social intelligence. This is what we did in this study, where the same measure was used on the AI represented by the large linguistic model (i.e., ChatGPT 4, Bing, and Google Bard). Our study showed important results regarding the superiority of AI in the field of SI.

The present findings showed that ChatGPT-4 completely outperformed the psychologists. Bing outperformed most of the psychologists at the bachelor’s level, while the differences in social intelligence were not significant between Bing and the psychologists at the doctoral level. Interestingly, the psychologists of doctoral holders significantly outperformed Google Bird, while the differences between Google Bird and undergraduate students were not statistically significant, meaning that Google Bird’s performance was equal to the performance of bachelor’s students on the SI scale.

The result showed that AI outperformed human SI measured by the same scale, and some of it was equal, as in the case of Google Bard, with a certain educational level, which is a bachelor’s degree, but it was lower than the level of doctoral. The human participants in this study were a group assumed to have high social intelligence, as many studies have found (Osipow and Walsh, 1973; Wood, 1984), as well as by looking at their average social intelligence measured in the current study compared to the hypothesized mean. By defining social intelligence as the ability to understand the needs, feelings, and thoughts of people in general and to choose wise behavior according to this understanding, it is practically assumed that this would reflected in the superiority of psychologists over the performance of AI. However, our results showed that the differences were of varying, with AI outperforming humans, especially ChatGPT-4, and psychologists with PhDs outperforming Google Bird, while the difference between humans and Ping was not statistically significant.

We believe that the poor performance of Google Bard in SI may be attributed to the date in which this research was conducted, as the Google Bard model was still new and in the early stages of its development, as Google may have been shocked and surprised by what the open AI had achieved. In addition, these results may be due to technical aspects related to the development of the algorithms used in Google Bard. We suggest conducting future studies to track the rapid development of these models, and the extent of their effects on the work of psychotherapists. Another pivotal point that must be pointed out is the ethical extent of the use of artificial intelligence in psychotherapy. Will AI models adhere to the ethics of psychotherapy? Will people want to receive psychotherapy provided by intelligent machines? What about the principles of confidentiality, honesty, empathy, acceptance, and client rights?…etc. These issues need further studies and guidelines for psychotherapists when using artificial intelligence services in counseling and psychotherapy.

What concerns us and those who need counseling and psychotherapy is that this study confirmed the superiority of AI models over humans. These results are partly consistent with the study of Elyoseph and Levkovich (2023) which evaluated the degree of social awareness among the large linguistic models of AI and the extent of the ability of these models to read human feelings and thoughts. They concluded that the ChatGPT was able to provide high-quality responses, and was empathic to patients’ questions, with results showing participants’ preference for chatbot responses over a doctor’s answers. Chatbot responses were also rated as significantly more sympathetic than doctor responses. Some studies that have examined AI for several purposes have indirectly demonstrated the ability of AI in several psychological and mental aspects. Some clients have reported preferring AI-powered assistants over psychotherapists because the assistants were able to deal with their feelings in a distinct and positive manner. It seems like these assistants were able to reflect on the clients’ emotions in a way that made them feel comfortable (Ayers et al., 2023; Bodroza et al., 2023; Eshghie and Eshghie, 2023; Haase and Hanel, 2023; Harel and Marron, 2023; Huang et al., 2023).

Another study by Open AI found that GPT4 outperformed humans in postgraduate admission tests in American universities. Literature has indicated that social intelligence is not only an ability in humans but also in artificial intelligence and large linguistic models based on dialog and chat in particular (Herzig et al., 2019). A recent qualitative shift has emerged in the field of artificial intelligence regarding the nature of human intelligence and its effects on the design and development of smart robots. This may create controversy, as social intelligence is added to the behavior of intelligent robots for practical purposes and to enable the robot to interact smoothly with other robots or people, that social intelligence may be a stepping-stone toward more human-like artificial intelligence (Dautenhahn, 2007; Guo et al., 2023).

These results confirm the superior ability of AI in SI, as measured by human psychological standards or personality trait tools, and through practical evaluation in conversations conducted between it and clients through the experiments (Herzig et al., 2019; Ayers et al., 2023; Bodroza et al., 2023; Eshghie and Eshghie, 2023; Harel and Marron, 2023).

However, there are references in the literature to concerns and criticisms about AI, some of which relate to errors in diagnoses related to dangerous conditions such as suicide, errors of hallucinations, and fears of moral deviations that need adequate attention and controls in the future studies (Li et al., 2022; Elyoseph and Levkovich, 2023; Grodniewicz and Hohol, 2023). Research also has pointed to a lack of consistency in their responses on psychological measures (Chang et al., 2023), and others have argued that it was necessary to define his role in specific functions (Sedlakova and Trachsel, 2023).

These differences in results may deepen the debate about psychologists’ fears of losing their profession to artificial intelligence. Many researchers believe that these fears have accompanied humans during each industrial revolution and ultimately conclude that industrial development helps humans, reduces the less competent individuals, and creates new professions that deal with the new will emerge. Although the changes this time may be more severe, psychologists will not lose their profession, but its form will change in order to adapt to the new developments. The benefit will be much greater than the losses, and the psychologist must absorb the change, live with its rapid development, and contribute to its management.

As for ethical and professional concerns, researchers believe that they are legitimate and realistic concerns, but based on the development of technology throughout history, it is clear that fear accompanies a person for his profession and ethics. However, development continues and it becomes clear that the fears are exaggerated, then some professions or part of them disappear and humans continually adapt to these changes. For example, the printing machine disappeared and there were developments in the secretarial function through the use of computers instead of the printing machine, and cotton workers turned into machine managers. This is why specialists in psychology, psychotherapy and psychiatry recommend absorbing the wave by understanding artificial intelligence and its applications and making the most of this. Developments in counseling and psychotherapy.

Regarding to the ethical aspect, there are legitimate and notable concerns, so we propose multiple forms and sources of solutions to this problem, namely the enactment of laws, the development of algorithms that limit moral deviation during use, and protective programs such as forgery detectors… etc. Since development will pass and will not stop at the limits of our fears, psychotherapists and legislators will need to constantly think about solutions to problems that may affect the profession and its ethics.

In conclusion, the ChatGPT 4 and Bing models have higher social intelligence than psychologists in the bachelor’s and doctoral stages, whereas the Bard model is on par with psychologists in the bachelor’s category and is outperformed by psychologists in the doctoral stage. According to our results, AI models can be ranked according to their performance on the social intelligence scale from highest to lowest, respectively, as follows: ChatGPT 4, Bing, and finally Google Bard.

The results of the current study can be useful and used to guide psychotherapists in their dealings with clients. Research evaluating the performance of AI models on measures of SI and other aspects of personality is urgently needed to improve the uses of AI in psychotherapy and mental health care planning.

There are some limitations in this study. The sample to verify the psychometric properties of the Social Intelligence Scale was small and homogeneous, and this is a relative shortcoming. This procedure was an additional confirmation since the validity and reliability of the scale had been previously verified by Sufyan (1998). There is a need for future studies that verify validity in a more precise manner on a large sample and in other ways to verify reliability in a more diverse or more precise way.

The social intelligence of the artificial intelligence models was evaluated only once. We were not able to re-evaluate and compare the two evaluations after a period due to the rapid developments in AI applications, which will affect the consistency of results over time. We suggest future longitudinal studies to track changes over time as AI models evolve. We used a subscription version of Chat GPT-4, and free versions of Bing and Google Bird, a difference that may have affected the results given the features available in the paid models compared to the free versions that available to the general public.

It was difficult to obtain a large sample of psychologists in Saudi Arabia, and we relied instead on psychological counseling students at the bachelor’s and doctoral levels (there were no master’s programs at the time of preparation of the study). We realize that this sample does not represent psychotherapists in the Kingdom of Saudi Arabia. However, it provides a good picture of human performance compared to the performance of AI in the SI scale. On the other hand, the study’s sample is confined to male counseling psychology students from a single university. This limited and homogeneous group might not reflect the broader population of psychologists or the general population’s social intelligence. Therefore, additional studies with a more diverse and representative sample are needed.

Although the study used a simple and homogeneous sample, its results are an important indicator of the superiority of these industrial systems, even though they appeared a very short time ago as systems simulating human behavior, and it is an indicator of the rapid future development of these systems in the coming years. This study is one of the first studies in this field, as it highlights and documents a historical stage in time for the beginning of the real competition between humans and machines in mental development, and the competition between the systems themselves. The results of the current study is also an indicator of industrial development compared to humans, paving the way for future studies that follow up on these developments and competitions.

Future studies will need to address the limitations of the current study. Our findings provide essential evidence about the degree of social intelligence in AI models that can be evaluated by human standards. These results will have promising future applications in the fields of assessment, diagnosis, and psychotherapy.

It would be fair to point out that the current study evaluated the performance of three different artificial intelligence models and compared them with a reasonable-sized sample of psychologists. In addition, most previous studies did not focus on evaluating social intelligence in artificial intelligence models as much as they focused on evaluating emotional intelligence (for example, Elyoseph et al., 2023), which increases the importance of the current study.

## Data availability statement

The raw data supporting the conclusions of this article will be made available by the authors, without undue reservation.

## Ethics statement

The studies involving humans were approved by The Research Ethics Committee at King Khalid University. The studies were conducted in accordance with the local legislation and institutional requirements. The participants provided their written informed consent to participate in this study.

## Author contributions

NS: Conceptualization, Data curation, Investigation, Software, Supervision, Writing – original draft. FF: Conceptualization, Methodology, Project administration, Visualization, Writing – review & editing. SA: Conceptualization, Investigation, Visualization, Writing – review & editing. JM: Conceptualization, Data curation, Investigation, Writing – original draft.

## References

[ref1] Ayers J. W. Poliak A. Dredze M. Leas E. C. Zhu Z. Kelley J. B.. (2023). Comparing physician and artificial intelligence Chatbot responses to patient questions posted to a public social media forum. JAMA Intern. Med. 183, 589–596. doi: 10.1001/jamainternmed.2023.1838, PMID: 37115527 PMC10148230

[ref2] Bell D. (2019). “The coming of post-industrial society” in Social stratification, class, race, and gender in sociological perspective. 2nd ed (New York:Routledge), 805–817.

[ref3] Bodroza B. Dinic B. M. Bojic L. (2023). Personality testing of GPT-3: limited temporal reliability, but highlighted social desirability of GPT-3's personality instruments results. arXiv:2306.04308v2. doi: 10.48550/arXiv.2306.04308

[ref4] Brants T. Popat A. Xu P. Och F. J. Dean J. (2007). Large language models in machine translation. In: In Proceedings of the 2007 Joint Conference on Empirical Methods in Natural Language Processing and Computational Natural Language Learning (EMNLP-CoNLL) (pp. 858–867).

[ref5] Chang Y. Wang X. Wang J. Wu Y. Zhu K. Chen H.. (2023). A survey on evaluation of large language models. *arXiv*:2307.03109. doi: 10.48550/arXiv.2307.03109

[ref6] Das A. Selek S. Warner A. R. Zuo X. Hu Y. Keloth V. K.. (2022). Conversational bots for psychotherapy: a study of generative transformer models using domain-specific dialogues. In: Proceedings of the 21st Workshop on Biomedical Language Processing, 285–297, Dublin: Association for Computational Linguistics.

[ref7] Dautenhahn K. (2007). “A paradigm shift in artificial intelligence: why social intelligence matters in the design and development of robots with human-like intelligence” in 50 years of artificial intelligence. eds. Lungarella M. Iida F. Bongard J. Pfeifer R., Lecture Notes in Computer Science, vol. 4850 (Berlin, Heidelberg: Springer)

[ref8] de Mello F. L. de Souza S. A. (2019). Psychotherapy and artificial intelligence: a proposal for alignment. Front. Psychol. 10:263. doi: 10.3389/fpsyg.2019.00263, PMID: 30804863 PMC6378280

[ref9] Elyoseph Z. Hadar-Shoval D. Asraf K. Lvovsky M. (2023). ChatGPT outperforms humans in emotional awareness evaluations. Front. Psychol. 14:1199058. doi: 10.3389/fpsyg.2023.1199058, PMID: 37303897 PMC10254409

[ref10] Elyoseph Z. Levkovich I. (2023). Beyond human expertise: the promise and limitations of ChatGPT in suicide risk assessment. Front. Psychiatry 14:1213141. doi: 10.3389/fpsyt.2023.1213141, PMID: 37593450 PMC10427505

[ref11] Eshghie M. Eshghie M. (2023). ChatGPT as a therapist assistant: a suitability study. arXiv:2304.09873. doi: 10.48550/arXiv.2304.09873

[ref9001] Flint S. W. Piotrkowicz A. Watts K. (2022). Use of Artificial Intelligence to understand adults’ thoughts and behaviours relating to COVID-19. Perspect. Public Health. 142, 167–174. doi: 10.1177/1757913920979332, PMID: 33472547 PMC9047094

[ref12] Gottschalk L. A. (1999). The application of a computerized measurement of the content analysis of natural language to the assessment of the effects of psychoactive drugs. Methods Find. Exp. Clin. Pharmacol. 21, 133–138. doi: 10.1358/mf.1999.21.2.529240, PMID: 10327394

[ref13] Grodniewicz J. P. Hohol M. (2023). Waiting for a digital therapist: three challenges on the path to psychotherapy delivered by artificial intelligence. Front. Psychol. 14:1190084. doi: 10.3389/fpsyt.2023.1190084, PMID: 37324824 PMC10267322

[ref14] Guo B. Zhang X. Wang Z. Jiang M. Nie J. Ding Y.. (2023). How close is chatgpt to human experts? Comparison corpus, evaluation, and detection. arXiv:2301.07597. doi: 10.48550/arXiv.2301.07597

[ref15] Haase J. Hanel P. H. (2023). Artificial muses: generative artificial intelligence chatbots have risen to human-level creativity. arXiv:2303.12003. doi: 10.48550/arXiv.2303.12003

[ref16] Hagendorff T. Fabi S. (2023). Human-like intuitive behavior and reasoning biases emerged in language models--and disappeared in GPT-4. arXiv:2306.07622 3, 833–838. doi: 10.1038/s43588-023-00527-x, PMID: 38177754 PMC10766525

[ref17] Han N. Li S. Huang F. Wen Y. Su Y. Li L.. (2023). How social media expression can reveal personality. Front. Psych. 14:1052844. doi: 10.3389/fpsyt.2023.1052844, PMID: 36937737 PMC10017531

[ref18] Harel D. Marron A. (2023). Human or machine: reflections on Turing-inspired testing for the everyday. arXiv:2305.04312. doi: 10.48550/arXiv.2305.04312

[ref19] Herzig A. Lorini E. Pearce D. (2019). Social intelligence. AI & Soc. 34:689. doi: 10.1007/s00146-017-0782-8

[ref20] Horn R. L. Weisz J. R. (2020). Can artificial intelligence improve psychotherapy research and practice? Admin. Pol. Ment. Health 47, 852–855. doi: 10.1007/s10488-020-01056-9 32715430

[ref21] Hounshell D. (1984). From the American system to mass production, 1800–1932: The development of manufacturing technology in the United States. Johns Hopkins University Press, Baltimore: JHU Press.

[ref22] Huang F. Kwak H. An J. (2023). Is chatgpt better than human annotators? Potential and limitations of chatgpt in explaining implicit hate speech. arXiv:2302.07736. doi: 10.1145/3543873.3587368

[ref23] Kachur A. Osin E. Davydov D. Shutilov K. Novokshonov A. (2020). Assessing the big five personality traits using real-life static facial images. Sci. Rep. 10:8487. doi: 10.1038/s41598-020-65358-6, PMID: 32444847 PMC7244587

[ref24] Kopp S. Krämer N. (2021). Revisiting human-agent communication: the importance of joint co-construction and understanding mental states. Front. Psychol. 12:580955. doi: 10.3389/fpsyg.2021.580955, PMID: 33833705 PMC8021865

[ref25] Landers R. N. Behrend T. S. (2023). Auditing the AI auditors: a framework for evaluating fairness and bias in high stakes AI predictive models. Am. Psychol. 78, 36–49. doi: 10.1037/amp0000972, PMID: 35157476

[ref26] Lei L. Li J. Li W. (2023). Assessing the role of artificial intelligence in the mental healthcare of teachers and students. Soft. Comput. 1–11. doi: 10.1007/s00500-023-08072-5, PMID: 37362257 PMC10072038

[ref27] Li X. Li Y. Liu L. Bing L. Joty S. (2022). Is gpt-3 a psychopath? Evaluating large language models from a psychological perspective. arXiv:2212.10529. doi: 10.48550/arXiv.2212.10529

[ref28] Maksimenko V. A. Runnova A. E. Zhuravlev M. O. Protasov P. Kulanin R. Khramova M. V.. (2018). Human personality reflects spatio-temporal and time-frequency EEG structure. PLoS ONE 13:e0197642. doi: 10.1371/journal.pone.0197642, PMID: 30192756 PMC6128450

[ref29] Mokyr J. Strotz R. (1998). The second industrial revolution, 1870–1914. Stor. dell’Econ. Mond. 21945, 1–14.

[ref30] Morales S. Barros J. Echávarri O. García F. Osses A. Moya C.. (2017). Acute mental discomfort associated with suicide behavior in a clinical sample of patients with affective disorders: ascertaining critical variables using artificial intelligence tools. Front. Psych. 8:7. doi: 10.3389/fpsyt.2017.00007, PMID: 28210230 PMC5289061

[ref31] O'Dell J. W. Dickson J. (1984). Eliza as a "therapeutic" tool. J. Clin. Psychol. 40, 942–945. doi: 10.1002/1097-4679(198407)40:4<942::AID-JCLP2270400412>3.0.CO;2-D 6548229

[ref9002] OpenAI. (2023). GPT-4 technical report. doi: 10.48550/arXiv.2303.08774

[ref32] Osipow S. H. Walsh W. B. (1973). Social intelligence and the selection of counselors. J. Couns. Psychol. 20, 366–369. doi: 10.1037/h0034793

[ref33] Russell S. J. Norvig P. (2010). Artificial intelligence a modern approach. 3rd Edition, Prentice-Hall, Upper Saddle River: London.

[ref34] Sedlakova J. Trachsel M. (2023). Conversational artificial intelligence in psychotherapy: a new therapeutic tool or agent? Am. J. Bioeth. 23, 4–13. doi: 10.1080/15265161.2022.2048739 35362368

[ref35] Sharan N. N. Romano D. M. (2020). The effects of personality and locus of control on trust in humans versus artificial intelligence. Heliyon 6:e04572. doi: 10.1016/j.heliyon.2020.e04572, PMID: 32923706 PMC7475230

[ref36] Sufyan N. S. (1998). Social intelligence and social values and their relationship to psychosocial adjustment among psychology students at Taiz university. Unpublished doctoral dissertation University of Baghdad, Iraq.

[ref37] Thirunavukarasu A. J. Ting D. S. J. Elangovan K. Gutierrez L. Tan T. F. Ting D. S. W. (2023). Large language models in medicine. Nat. Med. 29, 1930–1940. doi: 10.1038/s41591-023-02448-8, PMID: 37460753

[ref38] Wood G. B. (1984). The accuracy of counselors’ first impressions. Dissertation abstracts international, 45(05), B.

[ref39] Woodnutt S. Allen C. Snowden J. Flynn M. Hall S. Libberton P.. (2023). Could artificial intelligence write mental health nursing care plans? J. Psychiatr. Ment. Health Nurs. 31, 79–86. doi: 10.110.1111/jpm.12965 37538021

[ref40] Zhai J. Barreto A. B. Chin C. Li C. (2005). User stress detection in human-computer interactions. Biomed. Sci. Instrum. 41, 277–282. PMID: 15850118

[ref41] Zhi S. Zhao W. Wang R. Li Y. Wang X. Liu S.. (2023). Stability of specific personality network features corresponding to openness trait across different adult age periods: a machine learning analysis. Biochem. Biophys. Res. Commun. 672, 137–144. doi: 10.1016/j.bbrc.2023.06.012, PMID: 37352602

